# Learning a Memory-Enhanced Multi-Stage Goal-Driven Network for Egocentric Trajectory Prediction

**DOI:** 10.3390/biomimetics9080462

**Published:** 2024-07-31

**Authors:** Xiuen Wu, Sien Li, Tao Wang, Ge Xu, George Papageorgiou

**Affiliations:** 1 Fujian Provincial Key Laboratory of Information Processing and Intelligent Control, School of Computer and Big Data, Minjiang University, Fuzhou 350108, China; 2 College of Computer and Data Science, Fuzhou University, Fuzhou 350108, China; 3 SYSTEMA Research Center, European University Cyprus, Nicosia 1516, Cyprus

**Keywords:** trajectory prediction, scene layout, memory bank, multi-stage goal generator

## Abstract

We propose a memory-enhanced multi-stage goal-driven network (ME-MGNet) for egocentric trajectory prediction in dynamic scenes. Our key idea is to build a scene layout memory inspired by human perception in order to transfer knowledge from prior experiences to the current scenario in a top-down manner. Specifically, given a test scene, we first perform scene-level matching based on our scene layout memory to retrieve trajectories from visually similar scenes in the training data. This is followed by trajectory-level matching and memory filtering to obtain a set of goal features. In addition, a multi-stage goal generator takes these goal features and uses a backward decoder to produce several stage goals. Finally, we integrate the above steps into a conditional autoencoder and a forward decoder to produce trajectory prediction results. Experiments on three public datasets, JAAD, PIE, and KITTI, and a new egocentric trajectory prediction dataset, Fuzhou DashCam (FZDC), validate the efficacy of the proposed method.

## 1. Introduction

**Figure 1:**
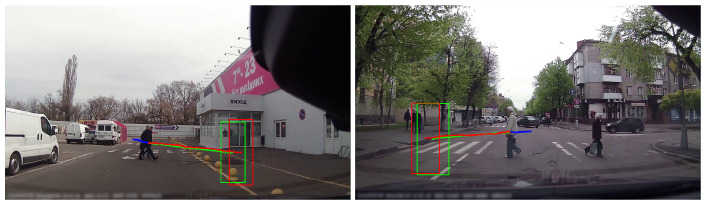
Example trajectory prediction results using our method on the JAAD dataset. The blue path represents the past trajectory, and the red, green paths, and bounding boxes represent the ground truths and predictions, respectively.

The prediction of future trajectories of surrounding agents in egocentric view is a crucial task in robotics and intelligent driving [1,2,3,4,5,6]. Such capacity plays an important role in improving the safety, reaction speed, and decision-making abilities of mobile robots and autonomous vehicles. As humans, we are able to quickly understand the behaviors of other moving agents such as pedestrians and cars in a dynamic scene, as shown in Figure 1. In particular, our perception of visual stimuli is influenced by the surrounding environment or context, and our prediction ability comes from adapting prior experiences to the current scene. Furthermore, our understanding of future movements is not limited to the trajectories of other agents, but also includes series of planned actions known as intentions [7].

In recent years, the rapid advancement of deep learning has greatly contributed to the accuracy of trajectory prediction. This progress has spawned a large amount of related research, leveraging a series of methods such as attention mechanisms [8,9,10,11], graph neural networks [12,13,14], generative models [15,16,17], and goal-driven networks [18,19,20,21,22] to better capture complex spatio-temporal relationships and understand movement patterns. However, state-of-the-art trajectory prediction methods either employ a parametric network that aims to encode the past trajectory and visual features [23,24,25,26], or a memory module that uses the past trajectory as a key to read future encodings from memory [27,28,29]. In addition, there is another line of research that adopts hybrid algorithms for trajectory prediction [30,31,32]. Unlike humans, the methods above lack an understanding of the overall scene layout and are therefore less interpretable, which is undesired in safety-critical applications such as mobile robots and autonomous vehicles.

Inspired by human perception, we seek to exploit the scene context for trajectory prediction in this work. However, it is challenging to design such a strategy for two reasons. Firstly, scene contexts are highly diverse, so it is important to build a memory module that is effective, efficient, and representative of typical scene layouts. In addition, what should be retrieved from previously observed scenes that are similar to the current scene remains an open question. Obviously, a single end goal may be insufficient, as the uncertainty in trajectory prediction increases with the lengthening of the prediction time span. Another possibility is to read previously observed trajectories directly (e.g., [28]). However, due to the diverse nature of scenes one would encounter, these trajectories have to be adapted to the current scene, which makes the complete previous trajectories redundant. In our work, we strike a balance between the two strategies above by applying the notion of intentions [7], i.e., a series of intermediate goals, to guide a multi-stage goal generator to produce multi-stage goals. More specifically, we first match a test scene to our scene layout memory to obtain the trajectories that belong to visually similar training scenes. Next, we use a past trajectory encoder to obtain a feature representation of the currently observed trajectory and compare it to trajectories from the visually similar scenes seen during training. The closest trajectory in the feature space is then retrieved, along with its future goals, to further go through a memory filter and then to be used as the input to the multi-stage goal generator with a backward decoder to obtain multi-stage goals. The multi-stage goals are then carried over to a forward decoder, which takes a conditional variational autoencoder (CVAE) to produce final trajectory predictions, which is commonly used in state-of-the-art trajectory prediction methods. Overall, our final model is semi-parametric in nature, comprising a parametric CVAE and bidirectional decoders as well as a non-parametric retrieval-based memory module. This design choice allows us to borrow the advantages of both types of methods, obtaining state-of-the-art performance in a number of egocentric vision-based trajectory prediction datasets.

This paper extends our previous work [26] in several important ways. Firstly, we build a scene layout memory bank to encode typical scene layouts, which is a significant new enhancement in our approach. Secondly, by comparing the test-time scene to the bank of typical scene layouts, we retrieve a set of intermediate goal features from similar scenes. This allows for a second step of matching the trajectories with a past trajectory encoder, using the currently observed trajectory as the key to retrieve the most similar trajectory seen during training. Thirdly, the retrieved trajectory is then used to query its own goal features to go through a memory filter and then a multi-stage goal generator. In terms of experimental evaluation, we conduct extensive experiments on two more datasets (i.e., KITTI and Fuzhou DashCam, FZDC) to demonstrate the superior performance of our method, and the new method also outperforms our previous work on the two existing datasets (i.e., JAAD and PIE). In particular, we propose a new egocentric trajectory prediction dataset, FZDC, that is publicly available to facilitate further research in this area.

In summary, our main contributions are outlined as follows:

- We present an innovative memory-enhanced multi-stage goal-driven network (ME-MGNet) for trajectory prediction. Given a test scene, we first propose a scene layout memory module inspired by human perception to borrow knowledge from previously seen similar scenes. The scene-level matching to previous experiences is followed by trajectory-level matching using the encoded features of past trajectories.
- Using the future goals associated with the retrieved past trajectory, we further build a joint reconstruction autoencoder that produces a series of goals. A memory filter is also presented to select if these goals are close enough to the current scenario.
- We integrate the above steps into a multi-stage goal generator that uses a backward decoder to produce multi-stage goals, and these goals are fed into a forward decoder that takes the output from a conditional autoencoder to obtain the final trajectory prediction results.
- The experimental evaluation results of four publicly available egocentric trajectory prediction datasets demonstrate the superior performance of our method compared to the state-of-the-art. We have created a new egocentric trajectory prediction dataset, FZDC, that will be made available to the research community. Moreover, ablation studies also verify the efficacy of the various components of the ME-MGNet.

The rest of this paper is organized as follows. Section 2 reviews recent literature in trajectory prediction; more specifically, goal-driven and memory-based methods, respectively. Then, Section 3 describes the proposed method in detail, followed by experimental evaluations in Section 4 and closing remarks in Section 5.

## 2. Related Work

### 2.1. Trajectory Prediction in Dynamic Video Scenes

At present, the majority of trajectory prediction methods operate using either an egocentric perspective or a bird’s-eye view. The egocentric camera perspective is generally regarded as the most intuitive viewpoint for observing the surrounding environment. Nonetheless, it presents additional challenges due to its restricted field of view and the effects of ego-motion. Many studies have tackled these challenges by converting the perspective into a bird’s-eye view with the aid of 3D sensors [33,34,35,36,37,38]. Although this approach is viable, it is prone to measurement errors and complications in multimodal data processing, particularly when using LiDAR and stereo sensors.

In this study, we focus on trajectory prediction from the egocentric perspective. A number of studies have been proposed to address this problem. For example, Bhattacharyya et al. [39] used Bayesian long short-term memory (LSTM) networks to model observation uncertainty, integrating these models with ego-motion to predict the distribution of potential future positions. Yagi et al. [23] used information such as pose, locations, scales, and past ego-motion to predict the future trajectory of a person. Chandra et al. [40] captured the interrelationships between nearby heterogeneous objects to predict trajectories. Yao et al. [41] proposed a multi-stream RNN encoder–decoder structure that captures both object location and appearance. Makansi et al. [42] estimated a reachability prior for objects based on the semantic map and projected this information into the future. Unlike the above methods, we propose a scene layout memory inspired by human perception, and we use the trajectories observed in visually similar scenes during training to aid in test-time trajectory prediction. In particular, we retrieve a set of intermediate goals from the memory, which is a representation that is neither overly simple nor redundant.

### 2.2. Goal-Driven Methods for Trajectory Prediction

In fact, goal-driven networks are widely used in trajectory prediction under the egocentric view. For instance, Mangalam et al. [19] proposed a long-range multi-modal trajectory prediction method by inferring distant trajectory endpoints. Rhinehart et al. [43] introduced a generative multi-agent forecasting method that learns on agent goals and models the relationships between individual agent goals. Zhao et al. [44] predict an agent’s potential future goal states by encoding its interactions with the environment and other agents, subsequently generating a trajectory state sequence conditioned on the goal. Yao et al. [24] propose a bidirectional decoder on the predicted goal to improve the accuracy of long-term trajectory prediction. Wang et al. [25] predict a series of stepwise goals at various temporal scales and integrate them into both encoders and decoders for trajectory prediction. The main contribution that sets our work apart from previous studies is that we predict future goals as intentions and build an intention memory bank of diverse scene layouts. Specifically, by comparing the currently observed trajectory to historical trajectories in the feature space, relevant intention features are retrieved from the memory bank to guide trajectory prediction.

### 2.3. Memory-Based Methods for Trajectory Prediction

A considerable body of research has introduced neural networks with memory functionality to address trajectory prediction. These networks can store and retrieve crucial information from sequential data, thereby improving trajectory prediction accuracy based on explicit memory information. One of the main advantages of these memory-based methods is their interpretability, and these methods are also considered complementary to the commonly used parametric neural networks. For example, Marchetti et al. [27] proposed a memory-augmented neural network to predict the trajectories of multiple targets. Specifically, they used recurrent neural networks to capture past and future trajectory features, leveraging a memory component to store and retrieve these features. Further, Xu et al. [28] proposed a sample-based learning framework incorporating a retrospective memory mechanism, storing samples from the training set into a pair of memory banks for matching relevant motion patterns during inference, thus improving the prediction accuracy. Furthermore, Huynh et al. [29] devised an adaptive learning framework that utilizes similarities between trajectory samples encountered during the testing process to enhance prediction accuracy. Different from the aforementioned methods, our work introduces the contextual information of the scene to build a scene layout memory bank, and we perform a two-level matching process (i.e., scene-level and trajectory-level) to retrieve a set of concise goal features for trajectory prediction. More importantly, we integrate the memory module into a conditional variational autoencoder, resulting in a semi-parametric final model that enjoys the benefits of both parametric and nonparametric methods.

### 2.4. Trajectory Prediction Using Clustering Methods

Lastly, as we will discuss in Section 3, our scene layout memory module involves a clustering step to encode the typical scene layouts. Therefore, we also include a brief discussion on trajectory prediction methods with a clustering component. For example, Akopov et al. [45] propose a cluster-based optimization of an evacuation process using a parallel bi-objective real-coded genetic algorithm (P-RCGA) based on the dynamic interactions of distributed processes that exchange the best potential decisions through a global population. Alam et al. [46] present a vessel trajectory prediction method that uses historical AIS data to cluster route patterns for each vessel type, thereby improving prediction accuracy. In addition, Sun et al. [47] propose a multimodal trajectory prediction method that involves a clustering step based on deep historical and future representations. Furthermore, Xue et al. [48] present a pedestrian trajectory prediction method using long short-term memory with route class clustering that captures pedestrian movement patterns. Unlike existing methods, the clustering that we perform in this work serves the purpose of identifying visually similar scene layouts, which has not been explored previously.

## 3. Memory-Enhanced Multi-Stage Goal-Driven Network

In this section, we present the proposed memory-enhanced multi-stage goal-driven network (ME-MGNet) in detail. Specifically, we first introduce our research objectives and hypotheses in Section 3.1, followed by presenting the formulation of the egocentric trajectory prediction problem in Section 3.2 and an overview to the proposed ME-MGNet in Section 3.3. Next, we describe the four main components of our methods, i.e., scene layout classification in Section 3.4, memory bank in Section 3.5, conditional variational autoencoder in Section 3.6, and multi-stage goal generator in Section 3.7. Finally, we present the overall learning objective and loss functions in Section 3.8.

### 3.1. Research Objectives and Hypotheses

The objective of egocentric trajectory prediction is to forecast the trajectory of a target object in a dynamic environment from a first-person perspective over a period of time. In the field of intelligent driving, accurate trajectory prediction enables vehicles to proactively respond to the movements of other vehicles and pedestrians on the road, facilitating safe and reasonable driving decisions. For mobile robots, trajectory prediction aids in more intelligent path planning, obstacle avoidance, and efficient task completion. In this paper, the main objective is to devise an effective strategy based on a memory module and multi-stage goal prediction for egocentric trajectory prediction in intelligent vehicles.

Based on the literature discussed in Section 2, we can readily see that memory modules and goal-driven models have been widely used for trajectory prediction. Unlike existing work, however, the two main research hypotheses in this paper are as follows. Firstly, we conjecture that building a memory of typical scene layouts would benefit trajectory prediction, because in two similar road scenarios, the behavior patterns of vehicles and pedestrians exhibit certain similarities. For example, at intersections, pedestrians often wait by the roadside before crossing the road. On straight roads, pedestrians tend to walk along the road or sidewalk. Therefore, scene-level appearance can be used to classify target trajectories with similar behavior patterns. Secondly, by predicting multiple intermediate goals, the trajectory prediction process can be more effectively guided, thereby reducing cumulative errors during the inference process and improving long-term prediction performance. This is similar to real-life scenarios, where road users typically need to plan a series of target positions to guide their direction before reaching their destination. Multiple intermediate goals are more detailed and can more accurately represent the movement intentions than a single goal.

### 3.2. Problem Formulation

The goal of trajectory prediction is to predict the future sequence of positions of a target within a scene based on the observed sequence of past positions. At time step *t*, we use $X_{t}=\left[X_{t}^{1},X_{t}^{2},\ldots ,X_{t}^{n}\right]$ to represent the past trajectories of *n* objects. In addition, $X_{t}^{i}=\left[{\bar{X}}_{t-\tau +1}^{i},{\bar{X}}_{t-\tau +2}^{i},\ldots ,{\bar{X}}_{t}^{i}\right]$ is the set of observation positions of object *i* in the past τ frames. Here, ${\bar{X}}_{t}^{i}=\left(x_{t}^{i},y_{t}^{i},w_{t}^{i},h_{t}^{i}\right)$ denotes the position and size of a bounding box, where $\left(x_{t}^{i},y_{t}^{i}\right)$ denotes the center coordinates and $\left(w_{t}^{i},h_{t}^{i}\right)$ represents the width and height of the bounding box, respectively. Given $X_{t}$, a predictor can be employed to predict the future trajectories $Y_{t}^{\prime }=\left[{Y_{t}^{\prime }}^{1},{Y_{t}^{\prime }}^{2},\ldots ,{Y_{t}^{\prime }}^{n}\right]$ for all *n* objects, where ${Y_{t}^{\prime }}^{i}=\left[{\hat{Y}}_{t+1}^{i},{\hat{Y}}_{t+2}^{i},\ldots ,{\hat{Y}}_{t+\rho }^{i}\right]$ represents the position of object *i* over the next ρ frames. The definition of ${\hat{Y}}_{t+1}^{i}=\left({\hat{x}}_{t+1}^{i},{\hat{y}}_{t+1}^{i},{\hat{w}}_{t+1}^{i},{\hat{h}}_{t+1}^{i}\right)$ is similar to $X_{t}^{i}$ and corresponds to a bounding box. We aim to design a model such that the predicted future trajectory $Y_{t}^{\prime }$ closely aligns with the actual future trajectory $Y_{t}$. The definition of $Y_{t}$ is similar to $Y_{t}^{\prime }$.

### 3.3. Overview

**Figure 2:**
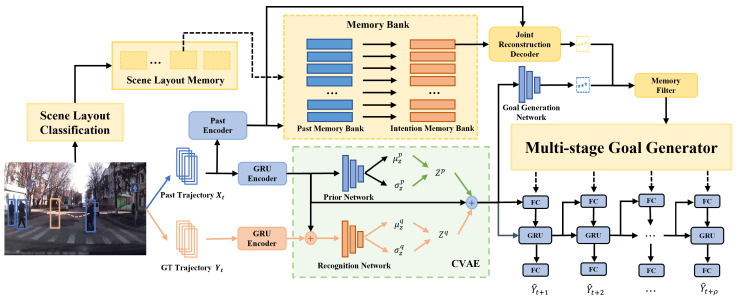
Overview of our ME-MGNet architecture. Arrows in orange, green, and black denote connections during training, inference, and both training and inference, respectively.

As shown in Figure 2, the memory-enhanced multi-stage goal-driven network (ME-MGNet) is mainly built around four key components: the scene layout classification, the memory bank, the conditional variational autoencoder, and the multi-stage goal generator. Before we move on to discuss the details for each of these components, let us begin with a high-level overview of how the ME-MGNet works.

The encoding stage of the network can be divided into two parts. In the first part, which is shown in the upper portion of Figure 2, we first search the scene layout memory for the scene class that is visually closest to the test scene with image-level features, and we use the past encoder to encode the past trajectory in order to retrieve the most similar entry from the past memory. Next, we map the past memory to its corresponding intention memory in the memory bank, then we concatenate the encoded past trajectory feature and the intention feature before feeding them into the joint reconstruction decoder to predict the motion intention of the target. In the second part, which is shown in the lower portion of Figure 2, following a popular practice (e.g., [19,24,25]), a conditional variational autoencoder (CVAE) is adopted as the encoder to learn the distribution of future trajectories. Different from existing approaches, we add a goal generation network that takes the latent representation *Z* from CVAE as input and outputs the motion intention represented by a series of several goals.

In the decoding stage of the network, the motion intention generated by the upper and lower branches in the encoding stage is fed into the memory filter, and the difference between the two is compared to decide whether to use the motion intention output from the memory module. Then, the output intention from the memory filter is used as the hidden state in the backward decoding process of the multi-stage goal generator. Finally, the forward decoder is connected to the hidden state vector of the backward decoder to predict the trajectory coordinates at each time step.

In the following, we outline the key steps of our method during inference. This will provide readers with a clear workflow of the ME-MGNet.

**Step 1**. At the current time step, given the input image, we use a feature extraction network in our scene layout classification module to obtain the scene layout features from the image.

**Step 2**. We compare the extracted scene-level features with the *K* scene layout features obtained using the K-means clustering algorithm during training to identify the most similar scene layout category. Subsequently, we select the corresponding memory bank from the scene layout memory according to the category.

**Step 3**. For each object in the scene, we encode its past trajectory using the past encoder to obtain feature $d_{t}$. Next, we compare $d_{t}$ with each of the features in the past memory bank $M_{p}$ in order to find the feature $k_{i}$ that is the most similar to $d_{t}$. Since the features in $M_{p}$ have a one-to-one correspondence to the features in the intention memory bank $M_{f}$, the future goal feature $v_{i}$ in $M_{f}$ is then retrieved, and together with $d_{t}$, they are fed into the joint reconstruction decoder to obtain the future goals ${\bar{G}}_{t}$.

**Step 4**. The past trajectory is used as the input into the conditional variational autoencoder (CVAE) module to obtain the predicted future goals ${\hat{G}}_{t}$.

**Step 5**. By comparing and integrating the goals obtained in Step 3 and Step 4, i.e., ${\bar{G}}_{t}$ and ${\hat{G}}_{t}$, the memory filter eliminates the ${\bar{G}}_{t}$ that deviates significantly from ${\hat{G}}_{t}$, and outputs the combined result $G_{t}^{\prime }$.

**Step 6**. The filtered future goals $G_{t}^{\prime }$ are input into the multi-stage goal generator to produce multi-stage goals, then they are combined with the forward decoding process of the CVAE to predict the trajectory coordinates at each time step.

### 3.4. Scene Layout Classification

Human perception almost always reflects an integration of top-down and bottom-up processes [49,50]. In particular, humans can quickly understand the gist of a scene, which is primarily a top-down process. Most existing methods in trajectory prediction, however, employ a bottom-up process and therefore lack an understanding of the overall appearance of the scene. In the context of trajectory prediction, the object movement patterns in similar scenes often have certain similarities. By classifying the scene layout, the environment in which the trajectory data are located can be divided into different scene categories to better learn the data distribution and improve the accuracy and robustness of trajectory prediction.

**Figure 3:**
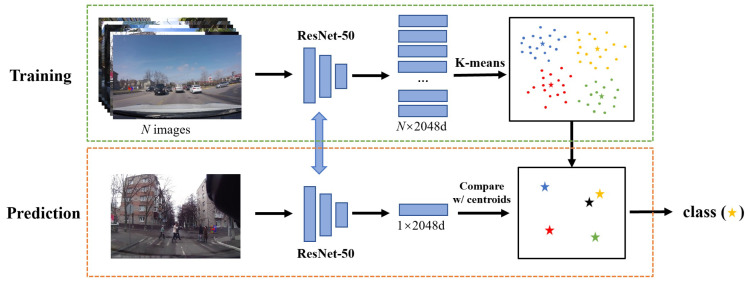
Illustration of the scene layout classification. The green dashed box represents the training process of the module, during which K-means clustering is performed on the scene-level image features. The orange dashed box represents the prediction process, during which the test image feature is compared to the cluster centroids to find the closest cluster as the classification result.

**Figure 4:**
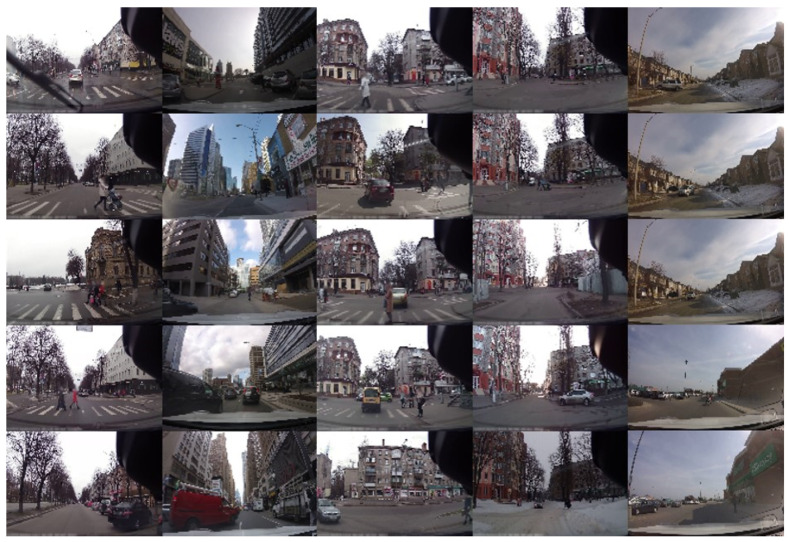
Scene layout clustering results. Each column shows example images from a cluster we obtained using K-means clustering, corresponding to a distinct scene layout.

In this paper, we use the K-means clustering algorithm [51] to perform clustering of road scenes in the training set and then classify similar scene layouts into the same category based on scene-level visual features. As shown in Figure 3, the overall procedure of the scene layout classification module in both training and prediction is shown. In the training stage, we use the ResNet-50 network [52], pre-trained on the ImageNet dataset [53], to extract the features from each image. Specifically, we utilize the 2048-dimensional features from the layer preceding the global average pooling layer of ResNet-50. This feature representation captures the high-level semantic information of the images while maintaining a relatively low dimensionality. Then, *K* feature vectors are randomly selected from the extracted features to serve as the initial cluster centroids. For each feature vector, its distances to the *K* cluster centroids are calculated, and the vector is assigned to the cluster with the closest centroid. The Euclidean distance is used to measure the distance between two feature vectors. Finally, the clusters are iteratively updated to cluster similar scene layouts into different categories. As shown in Figure 4, each column represents a cluster and corresponds to a specific scene layout. These clusters exhibit similar road layout features, such as crosswalks, through streets, and intersections.

After training, we can divide the images in the training set into *K* different clusters, each with its corresponding cluster centroid. Each cluster represents a typical scene layout. During the prediction stage, we continue to use the pre-trained ResNet-50 network to extract the 2048-dimensional features from the current test image. By comparing the test image features with the cluster centroids using the Euclidean distance, we can identify the most similar cluster and thereby determine the scene layout category of the current test image. In addition, we construct a memory bank for the trajectory data under each type of scene layout to learn the behavior patterns of objects in similar scenes. In other words, we build *K* different memory banks to handle trajectory prediction in various scenes, as further elaborated in the next section.

### 3.5. The Memory Bank

Next, let us move on to discuss the design of our memory bank. The primary goal of the memory bank is to establish a one-to-one correspondence between the past memory and the intention memory, effectively linking the past trajectory to the future intentions. Specifically, each memory bank is composed of a related past memory bank and intention memory bank. The past memory bank stores past trajectory features, while the intention memory bank stores corresponding future goal features, linking past trajectories with future goals through key–value pairs. Following existing literature, the term *intention* refers a series of *goals*, and each goal is a future coordinate of the target. Unlike the dense future trajectory, the series of goals is sparse and concise; for example, the total trajectory prediction span is 45 time steps on the JAAD dataset, and the goals correspond to the coordinates at the 15th, 30th, and 45th time steps, respectively. More formally, assume the past memory bank is $M_{p}=\left\{k_{i}|i=1,2,3,\cdots ,M\right\}$, where $k_{i}$ represents the instance at the *i*-th memory address, recording the past trajectory features extracted from the *i*-th training sample. Correspondingly, the intention memory bank is $M_{f}=\left\{v_{i}|i=1,2,3,\cdots ,M\right\}$, where $v_{i}$ represents the instance at the *i*-th memory address, recording the future goal features extracted from the *i*-th training sample. The two memory banks both store *M* instances, which is the number of total trajectories in a scene category. For each scene category, the total number of trajectories varies, and we omit the cluster membership subscript for notation simplicity. The past trajectory feature $k_{i}$ in the past memory bank is the key, and the corresponding value, which is the future goal feature $v_{i}$, can be found in the intention memory bank through the key.

**Figure 5:**
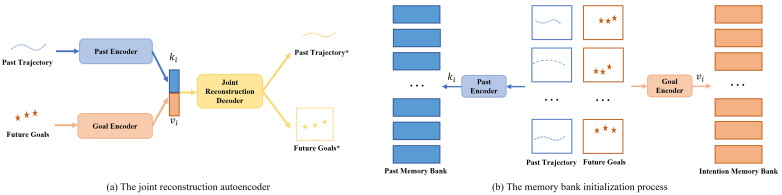
Memory bank initialization. We first train a joint reconstruction autoencoder, as shown in (**a**), and then use the two encoders in the joint reconstruction autoencoder to encode all the past trajectories and future goals in the scene cluster to initialize the memory bank, as shown in (**b**). The asterisks (*) indicate reconstruction results, and the stars indicate stage goals.

**The joint reconstruction autoencoder.** As shown in Figure 5a, to obtain the features in the memory bank, this paper proposes a joint reconstruction autoencoder to generate feature representations in the memory bank. At time *t*, two encoders are used to encode the past trajectory $X_{t}$ and the future goal $G_{t}$, respectively, to obtain the past trajectory feature $k_{i}$ and the future goal feature $v_{i}$. Here, a one-dimensional convolutional layer and a GRU unit are used as encoders to encode the temporal information. The encoding process can be written using the following formulation:

(1) $$k_{i}=GRU\left(Conv1D\left(X_{t}\right)\right)$$

(2) $$v_{i}=GRU\left(Conv1D\left(G_{t}\right)\right)$$

The decoder consists of a three-layer perceptron and takes the concatenated past trajectory features and future goal features as inputs, outputting the reconstructed past trajectory ${\tilde{X}}_{t}$ and future goals ${\tilde{G}}_{t}$. Unlike other memory models that construct feature representations, the future goals here are specifically composed of the positions of a series of three goals in the future trajectory. As a result, the reconstructed stage goals can more accurately describe the target’s behavioral intention. Therefore, the loss function of the joint reconstruction autoencoder can be defined as follows:

(3) $$L_{rec}={∥{\tilde{X}}_{t}-X_{t}∥}_{2}^{2}+\alpha {∥{\tilde{G}}_{t}-G_{t}∥}_{2}^{2}$$

where α is a weighting parameter. After training, the autoencoder can take the past trajectory and future goals as inputs, perform encoding and decoding operations, and thus reconstruct the past trajectory and future goal similar to the input. At test time, when the future goals are not available, we use the goal features retrieved from the memory bank, followed by the decoder part of the joint reconstruction autoencoder, to produce the predictions of future goals. This process is illustrated in the upper-right portion of Figure 2.

**Memory bank initialization.** The joint reconstruction autoencoder can map the past trajectory and the future goals into a meaningful pair of representations. As shown in Figure 5b, during the memory bank initialization process, the two encoders in Figure 5a are used to encode the past trajectory and the future goals, respectively. This process generates the past trajectory feature $k_{i}$ and the future goal feature $v_{i}$ in the memory bank, with the two features having a one-to-one correspondence in the form of key–value pairs.

**Memory retrieval.** After the memory bank initialization, it is also necessary to perform a retrieval operation based on the memory bank to obtain the future goal features corresponding to the past trajectories in the memory bank that are most similar to the current test data. This helps to better predict future trajectories by combining the encoded features. More specifically, the past encoder in Figure 5 is used to encode the observed test trajectory $X_{t}$ at the current time step *t* to obtain the encoded feature $d_{t}$. Next, the cosine similarity between the encoded feature $d_{t}$ and all the keys $k_{i}$ in the memory bank is calculated to perform retrieval and to find the past trajectory features that are the most similar. The similarity calculation formulation is given as follows:

(4) $$s_{m}=\frac{d_{t}k_{i}}{∥d_{t}∥∥k_{i}∥}$$

After calculating the pairwise similarities above, we sort the similarities of all keys, then select the key with the highest similarity and retrieve its corresponding value (i.e., one of the goal features $v_{i}$). This goal feature is concatenated with the observed trajectory feature $d_{t}$ and sent to the joint reconstruction decoder to output the future goals.

**Memory filter.** Relying solely on the trajectory information in the memory bank is not enough to cope with the diverse nature of scenes. In particular, test scenes may deviate greatly from the training scenes, and under such scenarios, the retrieved future goals may negatively impact the trajectory prediction. Therefore, we propose a memory filter to decide if the future goals ${\bar{G}}_{t}$ retrieved from the memory bank have a large deviation from the current test situation. If that is true, combining the predicted goals obtained from the memory often worsens the result. More specifically, our memory filter compares the prediction results ${\hat{G}}_{t}$ obtained by the goal generation network in the conditional autoencoder (described in Section 3.6) to the future goals generated by the joint reconstruction decoder in order to decide whether to discard the latter. This process is illustrated in the upper-right portion of Figure 2. When the goal ${\bar{G}}_{t}$ is filtered out, the filter outputs ${\hat{G}}_{t}$; otherwise, it outputs the average of the goals ${\bar{G}}_{t}$ and ${\hat{G}}_{t}$. Therefore, the formulation for the final output result $G^{\prime }$ of the filter is as follows:

(5) $$G_{t}^{\prime }={\hat{G}}_{t}+\frac{1}{2}s_{t}\left({\hat{G}}_{t},{\bar{G}}_{t}\right)\left({\bar{G}}_{t}-{\hat{G}}_{t}\right)$$

where $s_{t}\left({\hat{G}}_{t},{\bar{G}}_{t}\right)$ is the filtering function proposed in this paper, which estimates a binary score based on the predicted goals ${\hat{G}}_{t}$ obtained with the goal generation network and the predicted goals ${\bar{G}}_{t}$ retrieved from the memory bank. The formulation for calculating the score $s_{t}$ is given as follows:

(6) $$s_{t}=1\left(o_{t}>\delta \right),s_{t}\in \left\{0,1\right\}$$

where $1(o_{t}>\delta )$ represents an indicator function. When $o_{t}$ exceeds a pre-defined threshold of δ, the function outputs 1; otherwise, it outputs 0. In this paper, the threshold δ is set to 0.5 by default. The calculation formulation of $o_{t}$ is given as follows:

(7) $$\begin{array}{c} o_{t}=sig(FC\left(GRU\left(Conv1D\left({\hat{G}}_{t},{\bar{G}}_{t}\right)\right)\right) \end{array}$$

where sig and FC denote a sigmoid layer and a fully connected layer, respectively. We train the layers in the memory filter following the method proposed in the certainty-based selector in [29]. In summary, the final score $s_{t}$ is 0 or 1, which determines whether to filter out the goals predicted by the memory bank.

### 3.6. The Conditional Variational Autoencoder

Following recent work [19,24,25], we also use a conditional variational autoencoder (CVAE) to encode the past trajectory sequence to derive a latent variable *Z*, thereby learning and generating an approximate distribution of future trajectories. More specifically, our CVAE consists of the following modules: (1) A recognition network $Q_{\phi }\left(Z^{q}|X_{t},Y_{t}\right)$, which captures the correlation between the latent variable *Z* and the actual trajectory $Y_{t}$. (2) A conditional prior network $P_{\theta }\left(Z^{p}|X_{t}\right)$, which models the latent variable *Z* based on past observed trajectories $X_{t}$. (3) A goal generation network $P_{\omega }\left({\hat{G}}_{t}|X_{t},Z\right)$, which encodes input features and generates multi-stage goals. Here, ϕ,θ,ω denote the parameters of the corresponding networks. Each of these three networks consists of a three-layer multi-layer perceptron. In contrast to prior approaches, our goal generation network outputs several stage goals in the future trajectory, which more accurately represent the target’s behavioral intention and thus better guide the subsequent decoding process.

In the training stage, we encode the past trajectory $X_{t}$ and the ground truth future trajectory $Y_{t}$ separately using gated recurrent unit encoders (i.e., the GRU encoders in Figure 2), yielding feature vectors $h_{X}$ and $h_{Y}$, respectively. To capture the dependence information between the past trajectories and ground truth future trajectories, we concatenate the feature vectors $h_{X}$ and $h_{Y}$ and use them as inputs into the recognition network to predict the distribution mean $\mu_{z}^{q}$ and standard deviation $\sigma_{z}^{q}$ of future trajectories. The conditional prior network assumes that only $h_{X}$ is used to predict the distribution mean $\mu_{z}^{p}$ and standard deviation $\sigma_{z}^{p}$, without knowledge of the ground truth future trajectory. Next, we sample $Z^{q}$ from $N(\mu_{z}^{q},\sigma_{z}^{q})$, concatenate it with $h_{X}$, and ultimately use the goal generation network to obtain the goals ${\hat{G}}_{t}$ required by the memory filter. During the test phase, the ground truth future trajectories are not available. Therefore, unlike the training phase, $Z^{p}$ is sampled from $N(\mu_{z}^{p},\sigma_{z}^{p})$ to generate the goals ${\hat{G}}_{t}$.

### 3.7. The Multi-Stage Goal Generator

**Figure 6:**
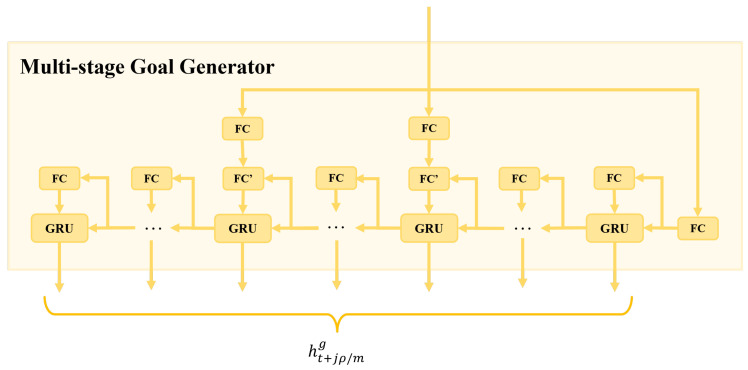
Detailed structure of the multi-stage goal generator. The stage goals output by the memory filter are used as inputs, guiding the generation of lower-level-stage goals in a top-down manner.

The network architecture of the multi-stage goal generator is shown in Figure 6. The stage goals output by the memory filter are used as inputs, guiding the generation of lower-level stage goals in a top-down manner. Specifically, the three input stage goals are passed through the fully connected layers to obtain three goal features. At the same time step, the three goal features are connected with the GRU hidden features in a backward recursive process of the lower layer, thereby guiding the generation of hidden features $h_{t+j\rho /m}^{g}$ of several stage goals from time t+ρ to t+1. The number of stage goal features *m* can be adaptively chosen according to the performance profile of the final model in a specific domain, which will be further discussed in Section 4. The output of the multi-stage goal generator can be defined as follows:

(8) $$\begin{array}{c} h_{t+j\rho /m}^{g}=f_{MSGE}(ReLU\left(FC\left(G_{t}^{\prime }\right)\right) \end{array}$$

Here, $G_{t}^{\prime }$ represents the stage goals produced by the memory filter. j={1,2,3,…,m} denotes the index of the j-th stage goal, ρ is the number of time steps to be predicted, and m∈[1,ρ] represents the division of the future trajectory into *m* stage goals. In addition, the function $f_{MSGE}$ denotes our multi-stage goal generator.

During the final decoding stage, the stage goal features obtained using the multi-stage goal generator are concatenated with the hidden state feature from the forward recursive inference to predict the final trajectory for that moment. It is worth noting that, due to the variable number of stage goal features output by the multi-stage goal generator, the connection between the generator and the forward recursive inference in the right portion of Figure 2 is represented with dashed lines.

### 3.8. Loss Functions

The overall loss function of the model in this paper consists of three parts: the trajectory prediction loss, the goal generation loss, and the KL divergence (KLD) loss. Specifically, the trajectory prediction loss quantifies the error between the model-predicted trajectory $Y_{t}^{\prime }$ and the true future trajectory $Y_{t}$. The future goal loss measures the error between the future goals $G_{t}^{\prime }$ produced by the memory filter and the true future goals $G_{t}$. Their calculation can be given as follows:

(9) $$L_{pred}={∥Y_{t}-Y_{t}^{\prime }∥}_{2}$$

(10) $$L_{goals}={∥G_{t}-G_{t}^{\prime }∥}_{2}$$

In addition, the KLD loss is used to measure the closeness between the distribution $N(\mu_{z}^{q},\sigma_{z}^{q})$ output by the recognition network of the CVAE module and the distribution $N(\mu_{z}^{p},\sigma_{z}^{p})$ output by the prior network in the same module. The total losses, including the KLD losses, are as follows:

(11) $$L_{total}=L_{pred}+L_{goals}+KLD(Z^{q}\;||\;Z^{p})$$

where $Z^{q}$ and $Z^{p}$ are latent variables sampled from distributions $N(\mu_{z}^{q},\sigma_{z}^{q})$ and $N(\mu_{z}^{p},\sigma_{z}^{p})$, respectively.

## 4. Experiments

### 4.1. Datasets

To better demonstrate the efficacy and robustness of the ME-MGNet, we evaluate our method using three public datasets, namely JAAD, PIE, and KITTI, and a new dataset, Fuzhou DashCam (FZDC), that we create in this paper:

**JAAD dataset [54].** The JAAD dataset is designed for studying trajectory prediction from an ego-vehicle perspective, focusing on the behavior of pedestrians and drivers on urban roads. It comprises 346 richly annotated short video clips (5–10 s long each) extracted from over 240 h of vehicle camera footage, with a video resolution of 1920×1080 and a video capture frequency of 30 Hz. The videos were collected at multiple locations in North America and Eastern Europe, covering various weather conditions, lighting conditions, and traffic scenarios. The JAAD dataset annotates video frames at a downsampled frequency of 30 Hz, providing a detection bounding box and a unique ID for each object. In accordance with the literature [55], we divide the 346 short video clips in the JAAD dataset into three groups in our experiments: a training set, a validation set, and a test set, containing 50%, 10%, and 40% of the data, respectively.

**PIE dataset [55].** The PIE dataset is also designed for studying motion prediction from a vehicle-mounted perspective. It contains over 6 h of vehicle-mounted video footage of typical traffic scenes, divided into 53 video sequences, each approximately 10 min long. Additionally, the dataset provides vehicle information (vehicle speed, direction, and GPS coordinates) from OBD sensors. All the video recordings are in high-definition format (1920×1080 pixels) at 30 frames per second. The PIE dataset annotates video frames at a downsampled frequency of 30 Hz, provides a detection bounding box and a unique target ID for each target, and includes rich scene and behavior annotations. Also, following the standard practice [55], the PIE dataset is divided into three groups for experiments: a training set, a validation set, and a test set, containing 50%, 10%, and 40% of the data, respectively.

**KITTI dataset [56].** The KITTI dataset is one of the most commonly used datasets in the field of autonomous driving and computer vision research. The dataset primarily contains various sensor data collected in real urban environments and can be used for tasks such as trajectory prediction, object detection and tracking, depth estimation, and optical flow estimation. Additionally, the dataset includes real image data from different scenes such as urban areas, rural areas, and highways, sampled and annotated at a frequency of 10 Hz, with an image resolution of 1238×374. Since the official KITTI dataset does not provide test set annotations, we use the training set from the object tracking task for trajectory prediction, which includes 21 video sequences, each ranging from 10 to 100 s long. Each sequence contains high-resolution RGB images, detection bounding boxes, unique target IDs, LiDAR point clouds, and the corresponding vehicle motion trajectories. We also divide the 21 video sequences into three groups: a training set, a validation set, and a test set, accounting for 50%, 10%, and 40% of the total data, respectively.

**Figure 7:**
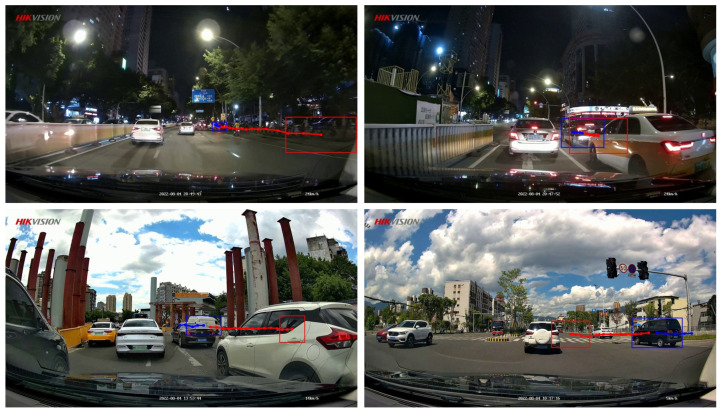
Example images from the Fuzhou DashCam (FZDC) dataset with trajectory annotations overlaid. The ground truth future trajectory is shown in red, and the past trajectory is shown in blue.

**FZDC dataset.** For trajectory prediction from the egocentric perspective, this paper proposes the Fuzhou DashCam (FZDC) dataset, which utilizes on-board cameras to record at 25 frames per second across multiple road sections in Fuzhou City, Fujian Province, China. The video resolution is 1280×720, capturing various road scenes such as urban main roads, highways, crosswalks, crossroads, and intersections. The collected and processed dataset contains a total of 66 video sequences, each lasting 20–30 s, with video frames annotated at a downsampled frequency of 10 Hz. Additionally, the dataset includes various object categories (e.g., vehicles, pedestrians, and cyclists). Each object is annotated with a bounding box, a unique ID, and a potential collision risk category, making the dataset suitable not only for trajectory prediction tasks but also for collision risk assessment tasks. An example of the trajectory annotation in the FZDC dataset is shown in Figure 7. The 66 video sequences in the dataset are divided into three groups: a training set, a validation set, and a test set, which account for 50%, 10%, and 40% of the total data volume, respectively. We note that the average driving behavior in China is somewhat different from the driving behaviors in more developed countries [57,58,59,60], and the FZDC dataset presents a unique and interesting challenge to accurate trajectory prediction. The FZDC dataset is publicly available from https://github.com/wxe999/FZDC_dataset (accessed on 29 June 2024) for research purposes.

### 4.2. Implementation Details

We conducted all experiments using a desktop server with an Ubuntu 20.04 OS, equipped with a 4.00 GHz Intel Core i9-9900KS CPU, 64 GB of RAM, and a single NVIDIA GeForce RTX 3090 GPU. Where our model uses gated recurrent units (GRU) as the backbone for both the encoder and decoder, the hidden size is set to 256. For the memory bank construction, the feature dimensions of the past memory bank and the intention memory bank are both set to 64. We use the Adam optimizer to train our model, starting with an initial learning rate of 0.001, which is dynamically adjusted according to the validation loss. We use the rectified linear unit (ReLU) as the activation function, and to mitigate overfitting, we integrate batch normalization and dropout layers. Our end-to-end optimization is conducted with a batch size of 128, and the training process concludes after 100 epochs.

### 4.3. Evaluation Metrics

In this paper, we follow the standard practice in recent work (e.g., [24,25]) and primarily assess the performance of our proposed approach using the mean squared error (MSE) between the positions of the upper-left and lower-right corners of the bounding box. The formulation for MSE is as follows:

(12) $$\begin{array}{c} \mathrm{MSE}=\frac{1}{n}\sum_{i=1}^{n}{\left(y_{i}-{\hat{y}}_{i}\right)}^{2} \end{array}$$

where *n* denotes the number of predicted objects, $y_{i}$ represents the ground truth, and ${\hat{y}}_{i}$ denotes the predicted value generated by the model.

Additionally, we also calculate two additional metrics for evaluation: the center mean squared error ($C_{MSE}$) and the center final mean squared error ($CF_{MSE}$). $C_{MSE}$ can measure the accuracy of the entire trajectory, while $CF_{MSE}$ only measures the accuracy of the endpoints of the trajectory. Both metrics are computed similarly to MSE, but we note that their calculations are based on the centroid of the bounding box. All the metrics are measured in pixels.

### 4.4. Results

**Quantitative comparison.** To ensure the reliability of the experimental results, we obtained the model output based on the average of three experiments. As presented in Table 1, we conduct a comparative analysis of our model against other state-of-the-art trajectory prediction algorithms on the JAAD and PIE datasets, with the best performance results under each metric indicated in bold. The proposed method achieved the best performance across all the metrics on the JAAD dataset. Compared to our previous work, MGNet ranked second, and our method had an average improvement of 3.2% in the MSE indicator and 3.1% and 3.5% in the $C_{MSE}$ and $CF_{MSE}$ indicators, respectively. Similarly, the proposed method also achieved the best performance under all metrics on the PIE dataset. The MGNet algorithm ranked second here as well, and our method showed an average improvement of 2.1% in terms of MSE and 2.3% and 4.2% in terms of $C_{MSE}$ and $CF_{MSE}$, respectively.

In addition, we perform performance evaluation using the KITTI and FZDC datasets. Based on the analysis in Table 2, it can be seen that the method in this paper achieves the best performance across all the metrics on the KITTI dataset. Compared with the MGNet algorithm, which ranks second in performance, ME-MGNet has an average performance improvement of 7.3% in terms of MSE and 14.3% and 10.2% in terms of $C_{MSE}$ and $CF_{MSE}$, respectively. In addition, ME-MGNet achieves the best performance for all the metrics on the FZDC dataset. Compared to the algorithm ranked second in performance, ME-MGNet has an average performance improvement of 2.6% in MSE and 3.1% and 3.0% in $C_{MSE}$ and $CF_{MSE}$, respectively. In summary, the performance comparison results on all four datasets clearly demonstrate the strong performance of ME-MGNet in improving the accuracy of trajectory prediction.

**Exploration study.** As shown in Table 3, we examine the performance impact on the JAAD and the PIE datasets by adjusting the number of stage goal features output by the multi-stage goal generator. In the table, outputting three stage goals means that the model does not use the multi-stage goal generator but only uses the three future stage goals output by the memory filter to guide trajectory generation. The results show that on the JAAD dataset, the best performance is obtained when we set the number of stage goals to 15, while on the PIE dataset, the best results are obtained when 9 stage goals are used. This indicates that the optimal number of output stage goal features depends on the dataset and requires further adjustment. Additionally, it can be observed from the table that when the multi-stage goal generator is not used, that is, when only three goal features are used, the results are the worst. This finding verifies the effectiveness of the multi-stage goal generator proposed in this paper.

In addition, we also explore the impact of adjusting the number of scene layout categories when performing K-means clustering on the prediction performance. The results are shown in Table 4. When the number of scene layout categories is 1, it means that the model does not classify the scene layout and only generates a single memory bank to assist in subsequent trajectory prediction. The experimental data show that on the JAAD dataset, the best results are obtained when the number of scene layout categories is set to 20, and then the prediction performance gradually decreases as the number of scene layout categories increases. On the PIE dataset, the best results are obtained when the number of scene layout categories is set to 25. Then, as the number of scene layout categories increases, the prediction performance gradually decreases. Through experimental observations, when the number of scene layout categories is too large, the originally similar scene layouts are further subdivided, resulting in less trajectory data in the memory bank of the corresponding scene layout. Consequently, it becomes impossible to retrieve effective goal features from the memory bank. This indicates that the number of scene layout categories should not be too large and needs to be further adjusted according to the dataset. Additionally, it is observed that when scene layout classification is not used, the results are the worst. This finding verifies the effectiveness of the scene memory bank proposed in this paper.

**Ablation study.** Next, we systematically remove various components from the model in the JAAD dataset to evaluate their impact on the experimental results. The results are shown in Table 5, where “BL” represents the baseline model, which only uses CVAE to encode and output a single goal to guide the forward recursive reasoning of trajectory prediction, without the scene memory bank module and the multi-stage goal generator. “MB” represents the memory bank module, and “MSG” represents the multi-stage goal generator. It can be clearly seen from the table that the inclusion of the scene memory bank module or the multi-stage goal generator both have positive impacts on the results. Among them, the performance is most significantly improved when the memory bank module is adopted, with an average performance improvement of 8.8% in MSE and 8.6% and 5.9% in $C_{MSE}$ and $CF_{MSE}$, respectively. When the multi-stage goal generator is used, the average performance is improved by 5.9% in terms of MSE and 7.1% and 4.1% in terms of $C_{MSE}$ and $CF_{MSE}$, respectively. Finally, the best results were achieved by combining both the memory bank and the multi-stage goal generator, with an average performance improvement of 13.8% in MSE and 13.2% and 10.9% in $C_{MSE}$ and $CF_{MSE}$, respectively. Based on the results above, we can clearly see the efficacy of the memory bank and the multi-stage goal generator proposed in this paper.

**Figure 8:**
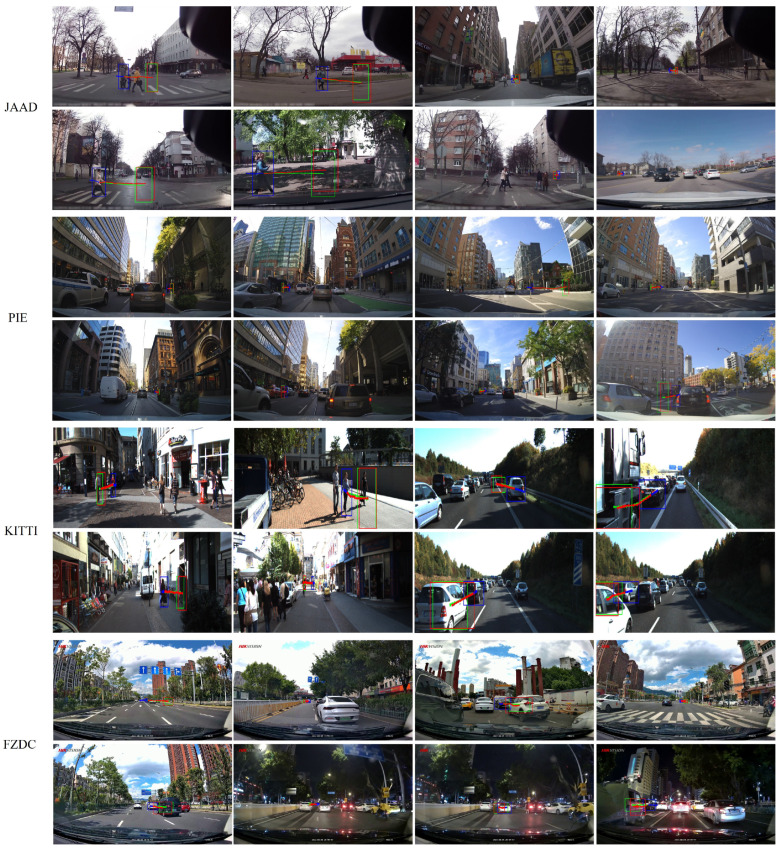
Qualitative results of trajectory prediction on JAAD, PIE, KITTI, and FZDC datasets. Red indicates ground truth, green indicates predictions, and blue indicates past trajectories. Best viewed in color.

**Qualitative results.** As shown in Figure 8, we present example prediction results on the JAAD, PIE, KITTI, and FZDC datasets to qualitatively demonstrate the prediction performance of our method. In the examples, the blue paths and bounding boxes represent the past trajectory, the red paths and bounding boxes represent the ground truth, and the green paths and bounding boxes represent the prediction results obtained using the ME-MGNet. It can be clearly observed that the test scenes contain real-life traffic scenarios, such as urban main roads, crosswalks, and intersections. The trajectories predicted by our method are largely accurate when compared to the ground truths, and they clearly capture the intentions of other road users. Furthermore, as the prediction time lengthens, the errors between the predicted trajectories and the ground truth increase, but it still maintains a relatively accurate prediction of the intentions rather than the exact trajectories, which is essential for road safety applications.

### 4.5. Discussion

In this paper, we propose two hypotheses based on existing work to improve model performance. First, to address the issue that existing trajectory prediction methods based on memory-augmented networks lack an understanding of the scene context, we redesign the memory bank within the memory-augmented network. Specifically, we divide the trajectory features in the memory bank into several clusters based on their scene layouts. The trajectory features stored in each cluster originate from similar road scenes and exhibit similar behavior patterns. This allows for accurate and efficient retrieval of historical trajectory information to guide future predictions. Second, unlike existing goal-driven networks that guide the generation of future trajectories by predicting a single long-term goal, we propose guiding future trajectory generation by predicting several stage-wise goals. Compared to a single long-term goal, multi-stage goals can more accurately reflect the behavioral intentions of the agent, thereby more precisely guiding the generation of future trajectories. Finally, we compare the proposed model with existing methods on four datasets, JAAD, PIE, KITTI, and FZDC, to verify its effectiveness. Among them, the first three datasets are widely used benchmarks for trajectory prediction, and the favorable experimental results demonstrate the superiority and generality of our method. We also create a new dataset, FZDC, to facilitate future research in this area. We conduct ablation experiments on the JAAD dataset, and the results successfully validated the two hypotheses we proposed. We note that our method can be readily integrated into existing methods based on a conditional autoencoder (e.g., [24,26]) and other trajectory prediction approaches lacking an understanding of the scene-level context to further enhance their performance.

**Figure 9:**
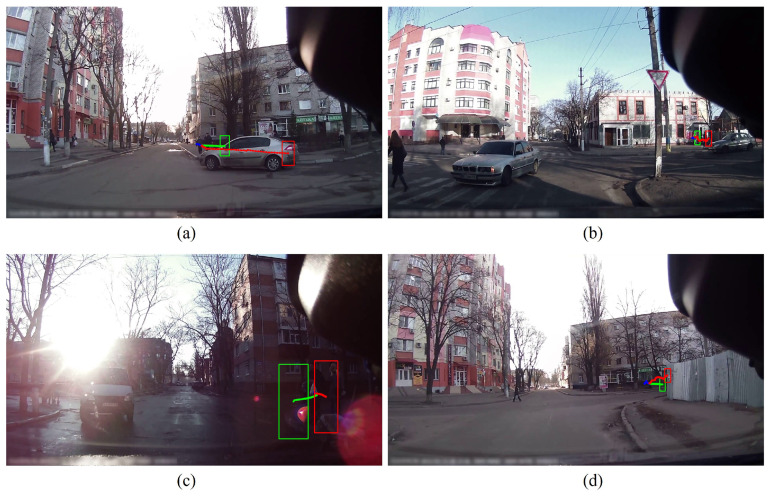
Examples of prediction failures on the JAAD dataset. Subfigures (**a**–**d**) are four typical failure examples. Red indicates ground truth, green indicates predictions, and blue indicates past trajectories. Best viewed in color.

**Limitations.** As shown in Figure 9, examples of prediction failures of the proposed method in some special cases are presented, highlighting areas for improvement in future research. Specifically, in Figure 9a, due to the small change in the past trajectory combined with the sudden acceleration of the vehicle, the change in the future trajectory suddenly increases, resulting in a large deviation between the prediction result and the actual situation. In Figure 9b, despite minimal changes in the past trajectory, the bumps and the increased speed of the vehicle cause the future trajectory to become abnormally fluctuated, making accurate prediction by the model almost impossible. The pedestrian’s distant nature further adds to the difficulty. In Figure 9c, the model incorrectly predicts that the pedestrian intends to cross the road, while the pedestrian decides not to do so. We note that, however, in the context of autonomous driving, it is crucial to place greater emphasis on the intentions of pedestrians crossing the street, as incorporating defensive driving can significantly enhance safety. In Figure 9d, due to vehicle bumps, both the past and future trajectories of the distant pedestrian exhibit large fluctuations, making it difficult for the model to accurately predict the trajectory. In general, typical challenging scenarios arise when there is a sudden change in the movement pattern of either the ego-vehicle or the other agents, as well as when dealing with small and distant objects.

## 5. Conclusions

In this work, we propose a memory-enhanced multi-stage goal-driven network (ME-MGNet) for egocentric trajectory prediction. By constructing a memory bank for different traffic scene layouts, the network can accurately retrieve the memory trajectory data related to the currently observed trajectory with a scene-level and a trajectory-level matching process. In addition, we propose a multi-stage goal generator that is able to adapatively select an appropriate number of stage goals for backward and forward decoding and future trajectory reasoning. The efficacy of ME-MGNet is validated on four public datasets, including the new Fuzhou DashCam dataset we create in this work to facilitate future research in this area.

## Figures and Tables

**Table 1:** Quantitative results on JAAD and PIE datasets. Lower values are better. The bold text indicates the best results.

Method	JAAD			PIE		
	MSE	$C_{\mathrm{MSE}}$	${\mathrm{CF}}_{\mathrm{MSE}}$	MSE	$C_{\mathrm{MSE}}$	${\mathrm{CF}}_{\mathrm{MSE}}$
	(0.5/1.0/1.5 s)	(1.5 s)	(1.5 s)	(0.5/1.0/1.5 s)	(1.5 s)	(1.5 s)
Linear [55]	223/857/2303	1565	6111	123/477/1365	950	3983
LSTM [55]	289/569/1558	1473	5766	172/330/911	837	3352
B-LSTM [39]	159/539/1535	1447	5615	101/296/855	811	3259
FOL-X [41]	147/484/1374	1290	4924	47/183/584	546	2303
PIEtraj [55]	110/399/1248	1183	4780	58/200/636	596	2477
PIEfull [55]	-	-	-	-/-/559	520	2162
BiTraP-D [24]	93/378/1206	1105	4565	41/161/511	481	1949
MGNet [26]	87/353/1132	1079	4452	37/145/474	445	1906
ME-MGNet	**84**/**343**/**1095**	**1046**	**4297**	**36**/**142**/**464**	**435**	**1825**

**Table 2:** Quantitative results on KITTI and FZDC datasets. Lower values are better. The bold text indicates the best results.

Method	KITTI			FZDC		
	MSE	$C_{\mathrm{MSE}}$	${\mathrm{CF}}_{\mathrm{MSE}}$	MSE	$C_{\mathrm{MSE}}$	${\mathrm{CF}}_{\mathrm{MSE}}$
	(0.5/1.0/1.5 s)	(1.5 s)	(1.5 s)	(0.5/1.0/1.5 s)	(1.5 s)	(1.5 s)
Linear [55]	161/399/982	827	2820	461/995/2133	1853	5238
LSTM [55]	121/361/905	786	2718	409/958/2077	1778	4978
B-LSTM [39]	134/350/848	718	2325	376/917/2103	1828	5178
BiTraP-D [24]	73/275/716	648	2158	325/838/1888	1460	4369
MGNet [26]	65/239/627	596	1926	301/815/1782	1402	4231
ME-MGNet	**64**/**230**/**569**	**511**	**1729**	**297**/**766**/**1760**	**1358**	**4101**

**Table 3:** The impact of varying number of stages in the multi-stage goal generator to the performance on the JAAD and PIE datasets. Lower values are better. The bold text indicates the best results.

Goals	JAAD			PIE		
	MSE	$C_{\mathrm{MSE}}$	${\mathrm{CF}}_{\mathrm{MSE}}$	MSE	$C_{\mathrm{MSE}}$	${\mathrm{CF}}_{\mathrm{MSE}}$
	(0.5/1.0/1.5 s)	(1.5 s)	(1.5 s)	(0.5/1.0/1.5 s)	(1.5 s)	(1.5 s)
3	88/361/1155	1096	4439	**35**/147/485	455	1996
9	86/352/1143	1082	4376	36/**142**/**464**	**435**	**1825**
15	**84**/**343**/**1095**	**1046**	**4297**	37/146/472	447	1945
45	89/358/1152	1092	4413	37/148/482	451	1964

**Table 4:** The impact of varying numbers of scene layout categories to the performance on the JAAD and PIE datasets. Lower values are better. The bold text indicates the best results.

K	JAAD			PIE		
	MSE	$C_{\mathrm{MSE}}$	${\mathrm{CF}}_{\mathrm{MSE}}$	MSE	$C_{\mathrm{MSE}}$	${\mathrm{CF}}_{\mathrm{MSE}}$
	(0.5/1.0/1.5 s)	(1.5 s)	(1.5 s)	(0.5/1.0/1.5 s)	(1.5 s)	(1.5 s)
1	88/375/1199	1120	4623	40/150/491	463	2031
10	87/359/1145	1092	4559	36/151/492	460	1976
15	84/344/1096	1062	4355	37/145/474	445	1906
20	84/**343**/**1095**	**1046**	**4297**	36/144/469	442	1895
25	**83**/345/1104	1061	4379	**36**/**142**/**464**	**435**	**1825**
30	85/352/1120	1071	4474	37/144/472	443	1920
35	84/358/1136	1083	4525	37/147/487	458	2015

**Table 5:** Ablation study of our method on JAAD datasets. Lower values are better. BL: the baseline model, MB: memory bank module, MSG: multi-stage goal generator. The checkmarks indicate if the corresponding components of our method are activated. The bold text indicates the best results.

BL	MB	MSG	MSE	$C_{\mathrm{MSE}}$	${\mathrm{CF}}_{\mathrm{MSE}}$
			(0.5/1.0/1.5 s)	(1.5 s)	(1.5 s)
✓			98/400/1269	1205	4823
✓	✓		90/365/1157	1101	4537
✓		✓	88/375/1199	1120	4623
✓	✓	✓	**84**/**343**/**1095**	**1046**	**4297**

## Data Availability

The JAAD dataset is available from https://data.nvision2.eecs.yorku.ca/JAAD_dataset/. The PIE dataset is available from https://data.nvision2.eecs.yorku.ca/PIE_dataset/. The KITTI dataset is available from https://www.cvlibs.net/datasets/kitti/. The FZDC dataset is available from https://github.com/wxe999/FZDC_dataset. Accessed on 30 July 2024.

## References

[1] Korbmacher R., Tordeux A. (2022). Review of pedestrian trajectory prediction methods: Comparing deep learning and knowledge-based approaches. IEEE Trans. Intell. Transp. Syst..

[2] Huang Y., Du J., Yang Z., Zhou Z., Zhang L., Chen H. (2022). A survey on trajectory-prediction methods for autonomous driving. IEEE Trans. Intell. Veh..

[3] Mozaffari S., Al-Jarrah O.Y., Dianati M., Jennings P., Mouzakitis A. (2020). Deep learning-based vehicle behavior prediction for autonomous driving applications: A review. IEEE Trans. Intell. Transp. Syst..

[4] Wang R., Wang M., Zhang Y., Zhao Q., Zheng X., Gao H. (2023). Trajectory Tracking and Obstacle Avoidance of Robotic Fish Based on Nonlinear Model Predictive Control. Biomimetics.

[5] Romero-Sorozábal P., Delgado-Oleas G., Laudanski A.F., Gutiérrez Á., Rocon E. (2024). Novel Methods for Personalized Gait Assistance: Three-Dimensional Trajectory Prediction Based on Regression and LSTM Models. Biomimetics.

[6] Zuo W., Gao J., Liu J., Wu T., Xin X. (2024). Whole-body dynamics for humanoid robot fall protection trajectory generation with wall support. Biomimetics.

[7] Locke E.A., Latham G.P. (1990). A Theory of Goal Setting & Task Performance.

[8] Huang Y., Bi H., Li Z., Mao T., Wang Z. Stgat: Modeling spatial-temporal interactions for human trajectory prediction. Proceedings of the IEEE/CVF International Conference on Computer Vision.

[9] Sadeghian A., Kosaraju V., Sadeghian A., Hirose N., Rezatofighi H., Savarese S. Sophie: An attentive gan for predicting paths compliant to social and physical constraints. Proceedings of the IEEE/CVF Conference on Computer Vision and Pattern Recognition.

[10] Yu C., Ma X., Ren J., Zhao H., Yi S. (2020). Spatio-temporal graph transformer networks for pedestrian trajectory prediction. Computer Vision–ECCV 2020: Proceedings of the 16th European Conference, Glasgow, UK, 23–28 August 2020, Proceedings, Part XII 16.

[11] Chiara L.F., Coscia P., Das S., Calderara S., Cucchiara R., Ballan L. Goal-driven self-attentive recurrent networks for trajectory prediction. Proceedings of the IEEE/CVF Conference on Computer Vision and Pattern Recognition.

[12] Sun J., Jiang Q., Lu C. Recursive social behavior graph for trajectory prediction. Proceedings of the IEEE/CVF Conference on Computer Vision and Pattern Recognition.

[13] Mohamed A., Qian K., Elhoseiny M., Claudel C. Social-stgcnn: A social spatio-temporal graph convolutional neural network for human trajectory prediction. Proceedings of the IEEE/CVF Conference on Computer Vision and Pattern Recognition.

[14] Shi L., Wang L., Long C., Zhou S., Zhou M., Niu Z., Hua G. SGCN: Sparse graph convolution network for pedestrian trajectory prediction. Proceedings of the IEEE/CVF Conference on Computer Vision and Pattern Recognition.

[15] Gupta A., Johnson J., Fei-Fei L., Savarese S., Alahi A. Social gan: Socially acceptable trajectories with generative adversarial networks. Proceedings of the IEEE Conference on Computer Vision and Pattern Recognition.

[16] Liang R., Li Y., Li X., Tang Y., Zhou J., Zou W. Temporal pyramid network for pedestrian trajectory prediction with multi-supervision. Proceedings of the AAAI Conference on Artificial Intelligence.

[17] Lee N., Choi W., Vernaza P., Choy C.B., Torr P.H., Chandraker M. Desire: Distant future prediction in dynamic scenes with interacting agents. Proceedings of the IEEE Conference on Computer Vision and Pattern Recognition.

[18] Albrecht S.V., Brewitt C., Wilhelm J., Gyevnar B., Eiras F., Dobre M., Ramamoorthy S. (2021). Interpretable goal-based prediction and planning for autonomous driving. Proceedings of the 2021 IEEE International Conference on Robotics and Automation (ICRA).

[19] Mangalam K., Girase H., Agarwal S., Lee K.H., Adeli E., Malik J., Gaidon A. (2020). It is not the journey but the destination: Endpoint conditioned trajectory prediction. Computer Vision–ECCV 2020: Proceedings of the 16th European Conference, Glasgow, UK, 23–28 August 2020, Proceedings, Part II 16.

[20] Deo N., Trivedi M.M. (2020). Trajectory forecasts in unknown environments conditioned on grid-based plans. arXiv.

[21] Dendorfer P., Osep A., Leal-Taixé L. Goal-gan: Multimodal trajectory prediction based on goal position estimation. Proceedings of the Asian Conference on Computer Vision.

[22] Mangalam K., An Y., Girase H., Malik J. From goals, waypoints & paths to long term human trajectory forecasting. Proceedings of the IEEE/CVF International Conference on Computer Vision.

[23] Yagi T., Mangalam K., Yonetani R., Sato Y. Future person localization in first-person videos. Proceedings of the IEEE Conference on Computer Vision and Pattern Recognition.

[24] Yao Y., Atkins E., Johnson-Roberson M., Vasudevan R., Du X. (2021). Bitrap: Bi-directional pedestrian trajectory prediction with multi-modal goal estimation. IEEE Robot. Autom. Lett..

[25] Wang C., Wang Y., Xu M., Crandall D.J. (2022). Stepwise goal-driven networks for trajectory prediction. IEEE Robot. Autom. Lett..

[26] Wu X., Wang T., Cai Y., Liang L., George P. A Multi-Stage Goal-Driven Network for Pedestrian Trajectory Prediction. Proceedings of the 2024 International Conference on Computer Vision, Image and Deep Learning (CVIDL).

[27] Marchetti F., Becattini F., Seidenari L., Bimbo A.D. Mantra: Memory augmented networks for multiple trajectory prediction. Proceedings of the IEEE/CVF Conference on Computer Vision and Pattern Recognition.

[28] Xu C., Mao W., Zhang W., Chen S. Remember intentions: Retrospective-memory-based trajectory prediction. Proceedings of the IEEE/CVF Conference on Computer Vision and Pattern Recognition.

[29] Huynh M., Alaghband G. Online Adaptive Temporal Memory with Certainty Estimation for Human Trajectory Prediction. Proceedings of the IEEE/CVF Winter Conference on Applications of Computer Vision.

[30] Zhong X., You Z., Cheng P. (2023). A hybrid optimization algorithm and its application in flight trajectory prediction. Expert Syst. Appl..

[31] Li S., Gu Q., Gong W., Ning B. (2020). An enhanced adaptive differential evolution algorithm for parameter extraction of photovoltaic models. Energy Convers. Manag..

[32] Chaulwar A., Botsch M., Utschick W. (2016). A hybrid machine learning approach for planning safe trajectories in complex traffic-scenarios. Proceedings of the 2016 15th IEEE International Conference on Machine Learning and Applications (ICMLA).

[33] Chai Y., Sapp B., Bansal M., Anguelov D. (2019). Multipath: Multiple probabilistic anchor trajectory hypotheses for behavior prediction. arXiv.

[34] Gao J., Sun C., Zhao H., Shen Y., Anguelov D., Li C., Schmid C. Vectornet: Encoding hd maps and agent dynamics from vectorized representation. Proceedings of the IEEE/CVF Conference on Computer Vision and Pattern Recognition.

[35] Salzmann T., Ivanovic B., Chakravarty P., Pavone M. (2020). Trajectron++: Dynamically-feasible trajectory forecasting with heterogeneous data. Computer Vision–ECCV 2020: Proceedings of the 16th European Conference, Glasgow, UK, 23–28 August 2020, Proceedings, Part XVIII 16.

[36] Song H., Luan D., Ding W., Wang M.Y., Chen Q. Learning to predict vehicle trajectories with model-based planning. Proceedings of the Conference on Robot Learning.

[37] Zhou Z., Ye L., Wang J., Wu K., Lu K. Hivt: Hierarchical vector transformer for multi-agent motion prediction. Proceedings of the IEEE/CVF Conference on Computer Vision and Pattern Recognition.

[38] Aydemir G., Akan A.K., Güney F. Adapt: Efficient multi-agent trajectory prediction with adaptation. Proceedings of the IEEE/CVF International Conference on Computer Vision.

[39] Bhattacharyya A., Fritz M., Schiele B. Long-term on-board prediction of people in traffic scenes under uncertainty. Proceedings of the IEEE Conference on Computer Vision and Pattern Recognition.

[40] Chandra R., Bhattacharya U., Bera A., Manocha D. Traphic: Trajectory prediction in dense and heterogeneous traffic using weighted interactions. Proceedings of the IEEE/CVF Conference on Computer Vision and Pattern Recognition.

[41] Yao Y., Xu M., Choi C., Crandall D.J., Atkins E.M., Dariush B. (2019). Egocentric vision-based future vehicle localization for intelligent driving assistance systems. Proceedings of the 2019 International Conference on Robotics and Automation (ICRA).

[42] Makansi O., Cicek O., Buchicchio K., Brox T. Multimodal future localization and emergence prediction for objects in egocentric view with a reachability prior. Proceedings of the IEEE/CVF Conference on Computer Vision and Pattern Recognition.

[43] Rhinehart N., McAllister R., Kitani K., Levine S. Precog: Prediction conditioned on goals in visual multi-agent settings. Proceedings of the IEEE/CVF International Conference on Computer Vision.

[44] Zhao H., Gao J., Lan T., Sun C., Sapp B., Varadarajan B., Shen Y., Shen Y., Chai Y., Schmid C. Tnt: Target-driven trajectory prediction. Proceedings of the Conference on Robot Learning.

[45] Akopov A.S., Beklaryan L.A., Beklaryan A.L. (2020). Cluster-based optimization of an evacuation process using a parallel bi-objective real-coded genetic algorithm. Cybern. Inf. Technol..

[46] Alam M.M., Spadon G., Etemad M., Torgo L., Milios E. (2024). Enhancing short-term vessel trajectory prediction with clustering for heterogeneous and multi-modal movement patterns. Ocean Eng..

[47] Sun J., Li Y., Fang H.S., Lu C. Three steps to multimodal trajectory prediction: Modality clustering, classification and synthesis. Proceedings of the IEEE/CVF International Conference on Computer Vision.

[48] Xue H., Huynh D.Q., Reynolds M. (2020). PoPPL: Pedestrian trajectory prediction by LSTM with automatic route class clustering. IEEE Trans. Neural Netw. Learn. Syst..

[49] McClelland J.L., Rumelhart D.E. (1981). An interactive activation model of context effects in letter perception: I. An account of basic findings. Psychol. Rev..

[50] Palmer S.E. (1999). Vision Science: Photons to Phenomenology.

[51] Hartigan J.A., Wong M.A. (1979). Algorithm AS 136: A k-means clustering algorithm. J. R. Stat. Soc. Ser. C (Appl. Stat.).

[52] He K., Zhang X., Ren S., Sun J. Deep residual learning for image recognition. Proceedings of the IEEE Conference on Computer Vision and Pattern Recognition.

[53] Deng J., Dong W., Socher R., Li L.J., Li K., Fei-Fei L. (2009). Imagenet: A large-scale hierarchical image database. Proceedings of the 2009 IEEE Conference on Computer Vision and Pattern Recognition.

[54] Rasouli A., Kotseruba I., Tsotsos J.K. Are they going to cross? a benchmark dataset and baseline for pedestrian crosswalk behavior. Proceedings of the IEEE International Conference on Computer Vision Workshops.

[55] Rasouli A., Kotseruba I., Kunic T., Tsotsos J.K. Pie: A large-scale dataset and models for pedestrian intention estimation and trajectory prediction. Proceedings of the IEEE/CVF International Conference on Computer Vision.

[56] Geiger A., Lenz P., Urtasun R. (2012). Are we ready for autonomous driving? the kitti vision benchmark suite. Proceedings of the IEEE Conference on Computer Vision and Pattern Recognition (CVPR) 2012.

[57] Jie L., van Zuylen H.J., Chen Y., Lu R. (2012). Comparison of driver behaviour and saturation flow in China and the Netherlands. IET Intell. Transp. Syst..

[58] Wang L., Yu C., Zhang Y., Luo L., Zhang G. (2018). An analysis of the characteristics of road traffic injuries and a prediction of fatalities in China from 1996 to 2015. Traffic Inj. Prev..

[59] Hussain B., Sato H., Xiong S., Miwa T., Nguyen N.T., Morikawa T. (2019). Cross-cultural differences in aberrant driving behaviors: Comparison of Japanese, Chinese, and Vietnamese drivers. J. East. Asia Soc. Transp. Stud..

[60] Farooq D., Moslem S., Faisal Tufail R., Ghorbanzadeh O., Duleba S., Maqsoom A., Blaschke T. (2020). Analyzing the importance of driver behavior criteria related to road safety for different driving cultures. Int. J. Environ. Res. Public Health.

